# The Role of m6A Methylation in Tumor Immunity and Immune-Associated Disorder

**DOI:** 10.3390/biom14081042

**Published:** 2024-08-22

**Authors:** Siyu Mu, Kaiyue Zhao, Shanshan Zhong, Yanli Wang

**Affiliations:** 1 Department of Neurology, The First Affiliated Hospital of China Medical University, Shenyang 110000, China; musiyucathym@163.com (S.M.); sszhong@cmu.edu.cn (S.Z.); 2 Department of Hepatology, Beijing Tsinghua Changgeng Hospital, School of Clinical Medicine, Tsinghua University, Beijing 102218, China; zkya03623@btch.edu.cn; 3 Department of Infectious Diseases, The First Affiliated Hospital of China Medical University, Shenyang 110000, China

**Keywords:** N6-methyladenosine, RNA modification, immune cells, tumor microenvironment, tumorigenesis, immunotherapy

## Abstract

N6-methyladenosine (m6A) represents the most prevalent and significant internal modification in mRNA, with its critical role in gene expression regulation and cell fate determination increasingly recognized in recent research. The immune system, essential for defense against infections and maintaining internal stability through interactions with other bodily systems, is significantly influenced by m6A modification. This modification acts as a key post-transcriptional regulator of immune responses, though its effects on different immune cells vary across diseases. This review delineates the impact of m6A modification across major system-related cancers—including those of the respiratory, digestive, endocrine, nervous, urinary reproductive, musculoskeletal system malignancies, as well as acute myeloid leukemia and autoimmune diseases. We explore the pathogenic roles of m6A RNA modifications within the tumor immune microenvironment and the broader immune system, highlighting how RNA modification regulators interact with immune pathways during disease progression. Furthermore, we discuss how the expression patterns of these regulators can influence disease susceptibility to immunotherapy, facilitating the development of diagnostic and prognostic models and pioneering new therapeutic approaches. Overall, this review emphasizes the challenges and prospective directions of m6A-related immune regulation in various systemic diseases throughout the body.

## 1. Introduction

Epitranscriptomic mechanisms, particularly the regulation of messenger RNA (mRNA) through modifications, critically influence RNA expression by modulating many biological processes, including splicing, stability, translation, and localization [1,2,3]. Several studies highlight that aberrant epigenetic alterations correlate with a spectrum of diseases, ranging from cancers to immune and neurological disorders [4,5,6]. To date, over one hundred distinct RNA modifications have been identified across various organisms, including yeasts, plants, flies, viruses, and mammals [7,8,9,10,11]. Among these, N6-methyladenosine (m6A) emerges as the most prevalent internal modification in eukaryotic cells, playing a pivotal role in diverse cellular functions and contributing to numerous human diseases [12]. Recent advancements in m6A molecular quantification and sequencing techniques have enabled the mapping of m6A modification with high resolution and accuracy, thereby enhancing our understanding of its potential mechanisms within the transcriptome [13,14].

The immune system plays a crucial role in host protection, with key functions including immune prevention, immune stabilization, and immune surveillance. It identifies and neutralizes foreign entities and abnormal cells, eliminates pathogens, and maintains immune balance through sophisticated self-regulation. At present, it has been found that m6A modification can affect the biological characteristics of immune cells, including the regulation of hematopoietic stem cell (HSC) differentiation, participation in dendritic cell (DC)-driven innate immune response, and so on [15,16]. In addition, m6A modification is an integral mechanism in tumor immune regulation and immune evasion and contributes to various immune dysregulation disorders [17]. Exploring novel molecular pathways through which m6A modulates intercellular cooperation and its regulation within the tumor microenvironment could provide deeper insights into the role of immune regulation in human disease development.

In this review, we discussed the recent findings of m6A-related changes of the immune microenvironment in the initiation and progression of diseases and how they can influence the cancers of eight systems throughout the body as well as other immune diseases. The potential for targeting m6A methylation as an immune checkpoint and novel diagnostic therapeutic avenue in cancers and aging-related diseases holds promise.

## 2. M6A Methylation-Related Proteins: From Writing to Interpreting

**Figure 1:**
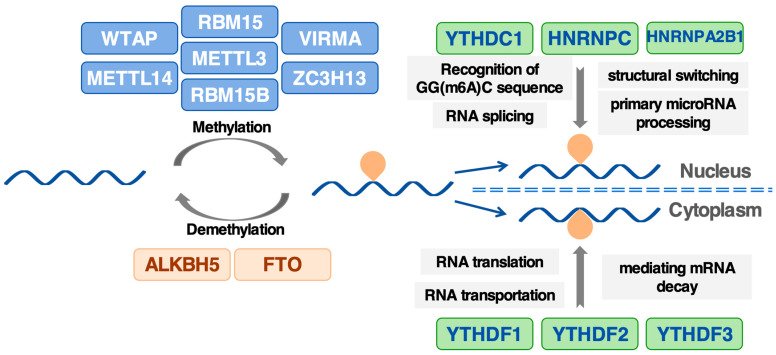
Overview of the m6A modification machinery in the mRNA lifecycle. m6A is installed by “writers” (such as WTAP, METTL14, METTL3, RBM15, RBM15B, VIRMA, and ZC3H13), removed by “erasers” (such as ALKBH5 and FTO), and recognized by “nuclear readers” (such as YTHDC1, HNRNPC, and HNRNPA2B1), and “cystoplasmic readers” (such as YTHDF1, YTHDF2, and YTHDF3).

M6A modification was discovered by Desrosiers et al. in 1974 and was later found to center on near stop codons and 3′ untranslated regions (3′ UTRs) with over three methylated sites in each mRNA [18,19]. It functions as a reversible dynamic mark in mRNAs adjusted by m6A regulators containing ‘writers’ (methyltransferases), ‘readers’ (signal converters), and ‘erasers’ (demethylases). The model of three m6A regulators is shown in Figure 1

The writers, which can methylate adenosine nucleotides of mRNAs via methyltransferase complex (MTC), mainly consist of methyltransferase-like 3 (METTL3), methyltransferase-like 14 (METTL14), RNA-binding motif protein 15/15B (RBM15/RBM15B), Wilms’ tumor 1-associating protein (WTAP), vir-like m6A methyltransferase associated (VIRMA, also termed KIAA1429) and zinc finger CCCH domain-containing protein 13 (ZC3H13). The central element of MTC is METTL3, featuring a domain for binding nucleic acids and actively participating in the catalytic process. Moreover, METTL3–METTL14 form the core of another complex in which the interaction between the substrate RNA and a conserved, positively charged interface of the METTL3–METTL14 heterodimer aligns the acceptor adenine moiety within the catalytic pocket of METTL3 [20]. WTAP functions as a regulatory element within the complex, significantly influencing gene expression and alternative splicing by targeting the localization of these complexes to nuclear speckles [21]. Similarly, RBM15/15B, while lacking catalytic activity, specifies DRACH sites for methylation in the transcriptome, acting in synergy with WTAP and METTL3 [22]. VIRMA and ZC3H13 also play crucial roles in the writer complex, interacting with other catalytic cofactors to facilitate RNA methylation [23,24].

The m6A readers include a suite of proteins capable of recognizing and binding to specific methylation sites on mRNAs, thereby modulating their fate. This group encompasses the YTH domain-containing family of proteins (YTHDF1/2/3, YTHDC1/2), the insulin-like growth factor 2 mRNA binding proteins (IGF2BP1/2/3), heterogeneous nuclear ribonucleoprotein A2B1 (HNRNPA2B1), and eukaryotic initiation factor 3 (eIF3). Within the nucleus, YTHDC1, HNRNPC, and HNRNPA2B1 mediate various posttranscriptional processes, such as the recognition of GG(m6A)C sequences, RNA splicing, structural switching, and primary microRNA processing [25,26]. In the cytoplasm, m6A-containing mRNAs are regulated by YTH family members, such as YTHDF1, YTHDF2, and YTHDF3, which selectively bind the methylated adenosine through a hydrophobic pocket [3,27]. YTHDF3, together with YTHDF1, cooperates in increasing mRNA translation efficiency through ribosomal proteins [28]. In addition, YTHDF3 could potentially be involved in RNA transportation and delivery as well as mediating mRNA decay by interacting with YTHDF2 and YTHDF1 in the relative early time point of the RNA lifecycle [29].

The erasers, tasked with removing methyl groups from m6A-modified RNAs, are composed of fat mass and obesity-associated protein (FTO) and alpha-ketoglutarate-dependent dioxygenase alkB homolog 5 (ALKBH5). FTO exhibits potent oxidative demethylation activity on prevalent m6A residues in RNA, demonstrated in vitro and partially localizing with nuclear speckles [30]. The substrates for FTO are considered to depend on different targets and still need to be further explored. ALKBH5 performs a similar demethylation function and is essential for processes affecting mRNA export and overall RNA metabolism [31]. These dynamic interactions provide insight into the regulation of biological processes via reversible m6A modifications of RNA, offering potential avenues for future research into epitranscriptomic regulation mechanisms.

## 3. M6A Modification in Immune Cells

The immune system coordinates responses to both internal and external stimuli through a diverse array of immune cell types, playing a critical role in the management of diseases, such as infections, autoimmune disorders, and cancers [32]. The modulation of immune responses by m6A methylation in the epitranscriptome is emerging as a pivotal mechanism across various aspects of immunity [5,33,34].

Hematopoietic stem and progenitor cells (HSPCs) are essential for the generation of blood cells, with m6A modifications significantly influencing their differentiation processes [35]. Recent evidence demonstrates that the disruption of METTL3 leads to decreased m6A levels in HSPCs, consequently altering cell proliferation and numbers. Furthermore, the degradation of mRNA mediated by YTHDF2 plays a crucial role in modulating the production of these progenitor cells [36]. Moreover, m6A modification can affect the energy metabolism of hematopoietic development and leukemia [37]. Gao et al. recently found that ALKBH5 could regulate the hematopoietic stem and progenitor cell metabolic switch from glycolysis to oxidative phosphorylation (OXPHOS) via control of the stability of metabolic gene transcripts [37].

Tumor-associated macrophages (TAMs) in TME are highly plastic and capable of switching their functions to inhibit or promote tumor progression in response to different environmental stimuli [38]. Macrophages, which are critical in disease pathogenesis, polarize into two distinct phenotypes: M1 (IFN-γ-producing) and M2 (IL-4-producing) [39,40]. m6A modification of STAT1 mRNA enhances its stability, thus promoting M1 polarization. Conversely, a reduction in METTL3 expression is associated with increased M2 polarization [41]. Recently, Yin et al. found that METTL3 in macrophages contributes to the regulation of tumor progression. Ablation of METTL3 in myeloid macrophages promotes tumor growth and metastasis [42]. A lack of METTL14 in macrophages can also inhibit the antitumor function of CD8+ T cells and promote tumor growth [43]. Furthermore, ALKBH5 interacts with DDX46 to modulate the demethylation of antiviral transcripts in infected macrophages, thereby impacting their antiviral response [44].

Dendritic cells (DCs), which are pivotal in initiating immune responses, exhibit modified antigen presentation capabilities due to m6A methylation [45]. METTL3 plays a crucial role in the maturation of DCs and subsequent T cell activation [15]. Mechanically, METTL3 enhances the translational efficiency of CD40, CD80, and toll/interleukin-1 receptor (TIR) structural domain interface protein (TIRAP), leading to the secretion of pro-inflammatory cytokines [15]. In contrast, the m6A reader YTHDF1 negatively modulates the antitumor immune response of DCs. YTHDF1 influences antigen degradation, thereby restricting DCs’ capacity to present tumor neoantigens and modulating their response to PD-L1 inhibitors [45].

T cells develop in the thymus, and as they mature, they migrate to peripheral organs and form the basis of the adaptive immune system [46]. T cells have an important protective role against viral infections and tumor cells and are key drivers of the cellular immune response [47]. Inhibition of METTL3 leads to a reduction in the level of m6A modification, which can have a major impact on T cells. This reduction affects the degradation of Socs mRNA and disrupts the IL-7/STAT5/SOCS signaling pathway, both of which are critical for T cell reprogramming and proliferation. B cells, vital for humoral immunity, are significantly influenced by deletions of METTL14 and YTHDF1/2, which impair early B cell development [48]. Specifically, METTL14 deletion severely hampers B cell development and function, while YTHDF1 and YTHDF2 distinctly affect early stages of B cell maturation [6]. Nuclephosmin 1 (NPM1) may affect the survival of B and NK cells by modulating glycolytic metabolism and YTHDF2-mediated m6A methylation in lung adenocarcinoma (LUAD) [49].

These findings underscore the complex role of m6A modification in immune cell differentiation, activation, and responses, providing valuable insights into the regulation of the immune system and the pathogenesis of diseases.

## 4. Aberrant m6A Modifications of Immune System Regulation in Human Cancer

**Figure 2:**
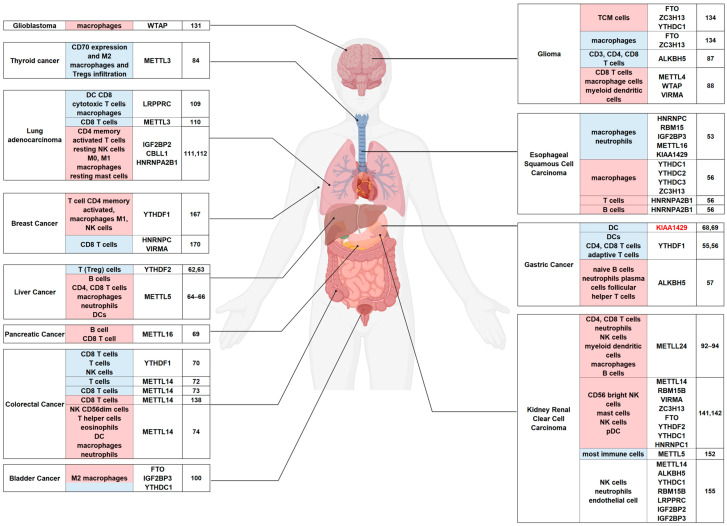
Immune-related tumor-promoting roles of N6-methyladenosine (m6A) modifiers. Aberrant m6A methylations caused overexpression (red) or downregulation (blue) of immune cells modified by different modifiers (writers, such as METTL3, METTL15, METTL16, and WTAP; readers, such as IGF2BP1–3 and YTHDC1; and erasers, such as FTO and ALKBH5) in different types of human cancers. KIAA1429 was discovered as a component of the m6A methyltransferase complex.

Here, we concluded the m6A modification models for human cancer in the order of different systems. (Figure 2, Table 1).

### 4.1. Digestive System

#### 4.1.1. Esophageal Cancer

Esophageal cancer (EC) is an aggressive malignancy, ranking as the eighth ninth most common malignant tumor worldwide [116]. Among its main histopathological subtypes, esophageal squamous cell carcinoma (ESCC) prevails over esophageal adenocarcinoma, posing a significant global health threat [117]. Recent studies have focused on understanding the interactions between single-cell m6A regulators and infiltrating immune cells in esophageal carcinoma, in which macrophages play a particularly important role [118]. Macrophages in ESCC can be categorized into two subtypes: M1-type and M2-type TAMs, which play opposite roles in tumor progression. The abundance of M1 macrophages negatively correlates with lymph node metastasis and the clinicopathological stage of ESCC; therefore, it is good for the prognosis of patients. On the other hand, M2 macrophages positively correlate with lymph node metastasis, tumor stage, and neovascularization density [50]. A significant correlation has been found between the expression of m6A regulators and the extent of macrophage infiltration. Mechanistically, METTL3 may attenuate inhibitory therapy with PD-1 by affecting macrophage reprogramming by regulating NAT10 in ESCC cells [119]. Moreover, in 2021, Wei et al. constructed a prognostic signature, which exhibited a notable negative correlation between the risk score and the levels of macrophage and neutrophil infiltration. They observed a substantial increase in PD-L1 expression within ESCC tissues, with a significant negative correlation noted with the expression levels of YTHDF2, METL14, and KIAA1429 [51]. Furthermore, HNRNPA2B1 exhibited higher expression in the tumor group of T cells, while HNRNPC showed increased expression in the tumor group of B cells, serving as a key mediator of PD-L1 expression in ESCC [52]. These findings emphasize the significant correlation between immune cells and m6A modifications in esophageal cancer and also provide a potential mechanism for EC PD-L1 immunotherapy.

#### 4.1.2. Gastric Cancer

Gastric cancer (GC) stands as a significant global health concern due to its aggressive nature [120]. Treatments such as chemotherapy, targeted therapy, and immunotherapy become necessary [121]. M6A regulators may contribute to the infiltration of immune cells and thus promote the antitumor immune ability.

YTHDF1,“reader” of m6A-modified mRNAs, plays a crucial role in cancer development and persistent neoantigen-specific antitumor immunity, has been identified as significantly deregulated, and is a prognostic marker associated with poor survival in GC [122]. According to a study by Bai et al., the loss of YTHDF1 enhances antitumor immunity through the facilitated recruitment of DCs, which activates both CD4+ and CD8+ T cells, leading to tumor remission. Furthermore, YTHDF1-associated immune infiltration in TME may be regulated by the p53 as well as JAK1/2-STAT1 pathway in tumor cells, contributing to increased sensitivity to antitumor immune responses [55,56]. These insights suggest that targeting YTHDF1 may offer a novel strategy to boost the efficacy of immunotherapy in GC by enhancing adaptive antitumor immunity.

Another m6A reader, ALKBH5, and its associated genes significantly influence the immune microenvironment in GC, particularly in stomach adenocarcinoma (STAD), although the findings are at times contradictory and ambiguous [123,124]. A study conducted by Ji et al. revealed that low protein levels of ALKBH5 in GC tissues are associated with the downregulation of SLC7A2 and CGB3. Importantly, tissue samples with higher ALKBH5 levels showed increased infiltration of naïve B cells, neutrophils, plasma cells, and follicular helper T cells [57]. Another study has demonstrated that upregulation of HSPA4 leads to reduced CD58 expression in GC cells through the HSPA4/ALKBH5/CD58 axis, which subsequently activates PD1/PDL1 pathways and impairs the cytotoxicity of CD8+ T cells, thereby facilitating immune evasion of GC cells [125]. These findings collectively underscore ALKBH5 as a promising noninvasive diagnostic and therapeutic biomarker for patients with gastric cancer.

The fate and function of m6A-methylated RNAs are primarily mediated through readers, including the YTH structural domain family of proteins (YTHDF1, YTHDF2, YTHDF3, and YTHDC1). Research conducted by Yu et al. and Zhang et al. revealed that YTHDF3 was identified as a promising prognostic marker linked to an unfavorable prognosis in patients with STAD [58,59]. This association is attributed to the activation of the PI3K/AKT pathway and various aspects of immune cell infiltration in tumors, including CD8+ T cells, macrophages, Tregs, major histocompatibility complex (MHC) molecules, and chemokines. Consequently, it may induce upregulation of PD-L1 and CXCL1, both critical components in the immune response to GC and its immunotherapeutic approaches [59].

The formation of m6A methylation is catalyzed by a methyltransferase complex composed of m6A writers in which KIAA1429 plays a key role. KIAA1429 is localized to nuclear speckles and has been demonstrated to recruit METTL3, METTL14, and WTAP, thereby facilitating regionally selective methylation [53]. Research has identified a significant negative correlation between KIAA1429 expression and the infiltration of invasive immune cells in the TME of GC, which is manifested through the suppression of immune activation pathways. Notably, a trend toward survival benefit has been observed in patients with low expression of KIAA1429 undergoing anti-PD-L1 immunotherapy (IMvigor210) [54].

#### 4.1.3. Liver Cancer

Hepatocellular carcinoma (HCC) represents one of the most prevalent malignant tumors and encompasses distinct subtypes, including hepatocellular carcinoma (HCC), intrahepatic cholangiocarcinoma (ICC), and mixed hepatocellular-cholangiocarcinoma (cHCC-CCA) [126]. Recent studies have identified m6A as a novel epigenetic modification that plays a crucial role in the progression of HCC [127]. M6A regulators play a bidirectional role in HCC. Some can promote immune cell infiltration and enhance antitumor immunity by activating certain signaling pathways, while others may influence TME to promote tumor proliferation and metastasis.

ALKBH5 was the second identified m6A demethylase, which has been shown to modulate mRNA export and RNA metabolism by reducing the m6A level in nuclear speckles [128]. Controversial expressions and functions have been reported in HCC for ALBKH5. Characterized as a tumor suppressor, ALBKH5 was found to be downregulated in HCC and showed tumor suppressor properties [60]. However, an investigation revealed that upregulated ALKBH5 enhances HCC cell proliferation, metastasis, and the recruitment of PD-L1-positive macrophages. Mechanistically, ALKBH5 activation of the JNK and ERK pathways, through upregulation of MAP3K8, modulates IL-8 expression and promotes macrophage recruitment, facilitating HCC cell proliferation and metastasis [61].Similarly, upregulated ALKBH5 in hepatocellular carcinoma stem cells was found to be a key effector associated with macrophage M2 polarization [129]. This evidence guides immunotherapy targets for HCC, which were further explored by Qiu et al., who demonstrated ALKBH5’s complex role in TME in ICC, where it upregulates PD-L1 on mononuclear/macrophage cells and decreases myeloid inhibitory cell populations. As evidence, both in vitro and in vivo studies show that tumor-derived ALKBH5 inhibits T cell expansion and cytotoxicity by sustaining PD-L1 expression [130].

Although previous studies also have found inconsistent functions in the occurrence and survival rates in HCC, nowadays the role of YTHDF2 in regulating Tregs underscores its potential as a therapeutic target in cancer immunotherapy [62,63]. The study conducted by Zhang and colleagues demonstrates that deletion of YTHDF2 in regulatory Tregs diminishes tumor growth, increases Treg apoptosis, and impairs the function of TME in murine models. Furthermore, elevated TNF signaling within TME has been shown to upregulate YTHDF2 expression, which subsequently modulates NF-κB signaling by targeting m6A-modified transcripts that encode the negative regulators of NF-κB [131].

METTL5 may serve as a promising target in antitumor immunotherapy, given its association with the increased expression of PD1 (PDCD1) and CTLA4 in HCC specimens due to its regulatory effects on TME [132]. Recent studies on METTL5 have focused on glucose and fat metabolism as well as immune-related regulation, where researchers recently found a positive correlation between METTL5 expression and the infiltration of B cells, CD8+ T cells, CD4+ T cells, macrophages, neutrophils, and DCs in liver cancer [64,65,66].

#### 4.1.4. Pancreatic Cancer

Pancreatic cancer (PC) is a leading cause of cancer-related mortality globally [133]. The immunosuppressive microenvironment within the tumor is a critical factor contributing to the adverse prognosis observed in patients at high risk for PC. Furthermore, the expression and regulation of immune checkpoint molecules are pivotal in modulating the tumor immune response [134,135]. METTL3 influences the expression of PD-L1, potentially through its interactions with the long non-coding RNA MALAT1 and the 5′-to-3′ exonuclease DCLRE1B in pancreatic cancer cells [67,68]. A research conducted by Lu L et al. highlights METTL16 as a significant new target for immunotherapy in pancreatic ductal adenocarcinoma (PDA), owing to its unique expression profiles across various driver gene mutations and its association with favorable prognostic outcomes. Importantly, METTL16 expression is positively associated with the infiltration of B cells and CD8+ T cells, as well as with the expression of immune checkpoints and cytokines [69]. These findings indicated that METTL16 is a potential immunotherapy target for PDA.

#### 4.1.5. Colorectal Cancer (CRC)

Colorectal cancer (CRC), characterized by a prevalent occurrence and a low, 5-year survival rate among metastatic patients, demonstrates promising responses to immune checkpoint blockade (ICB) [136]. M6A regulators such as YTHDF1, METTL3 and IGF2BP3 exhibit different effects by influencing immune cells, including the infiltration of cytotoxic T cells, and by regulating cytokine levels within the TME.

The recent discovery of YTHDF1 as a new potential biomarker of immunotherapy outcomes has the potential to distinguish patients who can benefit more from immune checkpoint blockade therapy [70]. Bao et al. elucidated that YTHDF1 contributes to the impairment of antitumor immunity through the m6A-p65-CXCL1/CXCR2 axis, thereby enhancing CRC progression. Notably, the expression of YTHDF1 exhibits a negative correlation with interferon-γ levels in TCGA-CRC and the infiltration of CD8+ T cells. Single-cell RNA sequencing (scRNA-seq) data further reveal a reduction in myeloid-derived suppressor cells (MDSCs) and an increase in cytotoxic T cells in YTHDF1-knockout tumors. Targeting YTHDF1 using CRISPR or VNPs-siYTHDF1 enhances the efficacy of anti-PD1 therapy in microsatellite instability—high (MSI-H) CRC and overcomes resistance in microsatellite-stable (MSS) CRC [71].

METTL3 and METTL14 antagonize immunotherapy by fostering immunosuppression within TME. Research shows that METTL14-mediated m6A levels demonstrate an inverse correlation with dysfunctional T cell abundance in CRC patients. Targeted deletion of the m6A methyltransferase METTL14 specifically in macrophages drives the differentiation of CD8+ T cells towards a dysfunctional phenotype, thereby compromising their efficacy in tumor elimination [43]. Moreover, Wang et al. demonstrated that METTL3 or METTL14 deficiency in tumors leads to an increase in cytotoxic tumor-infiltrating CD8+ T cells and an elevation in the secretion of IFN-γ, Cxcl9, and Cxcl10 within the tumor microenvironment in vivo. Mechanistically, the loss of METTL3 or METTL14 enhances the IFN-γ-Stat1-Irf1 signaling pathway by stabilizing Stat1 and Irf1 mRNA via Ythdf2. Furthermore, a negative correlation is observed between METTL3 or METTL14 and STAT1 in patients diagnosed with microsatellite-stable/mismatch repair-proficient CRC tumors [72]. Interestingly, METTL3 undergoes epigenetic modifications during CRC progression. Specifically, two lactoacylated modification sites are identified within the zinc finger domain of METTL3. Lactoacylated METTL3-mediated RNA m6A modification promotes the immunosuppressive inhibition of tumor-infiltrated bone myeloid cells (TIMs), thereby contributing to tumor immune evasion [137].

In CRC, ALKBH5 expression is diminished. However, ALKBH5 plays a pivotal role in fostering the infiltration of CD8+ T cells within the tumor microenvironment through the NF-κB-CCL5 axis. This mechanism serves to mitigate the malignant progression of CRC by restraining the proliferation, migration, and invasion of CRC cells [73]. Akin to METTL3, ALKBH5 also influences the response to anti-PD-1 therapy by regulating lactate levels and the accumulation of suppressive immune cells within the tumor microenvironment [138].

Several studies have suggested that IGF2BP3 holds promise as a prognostic biomarker in colon cancer patients [139,140]. IGF2BP3 expression exhibits a positive correlation with NK CD56dim cells, T helper cells, eosinophils, dendritic cells (DCs), and macrophage neutrophils, while showing a negative correlation with CD8 T cells. Intriguingly, individuals with elevated IGF2BP3 expression levels demonstrate enhanced responses to PD-1 and CTLA-4 therapies, hinting at the potential for novel personalized immunotherapy strategies [74,75].

#### 4.1.6. Gallbladder Cancer

Gallbladder cancer (GBC) stands out as the prevailing malignancy within the biliary tract, with adenocarcinoma representing its principal histological subtype [141]. As outlined by BaiX et al., the upregulation of YTHDF2 enhances the proliferation, tumor growth, migration, and invasion capabilities of GBC cells, while concurrently suppressing apoptosis both in vitro and in vivo. YTHDF2 acts by binding to the 3′-UTR of death-associated protein kinase 3 (DAPK3) mRNA, thereby promoting its degradation through an m6A-dependent mechanism. Notably, the YTHDF2/DAPK3 axis contributes to the development of resistance in GBC cells against gemcitabine treatment [142]. Furthermore, it was observed that IGF2BP3 expression levels were increased and positively associated with an aggressive phenotype [76]. Functionally, IGF2BP3 was found to stabilize CLDN4 mRNA through an m6A-dependent mechanism, consequently promoting macrophage immunosuppressive polarization and ultimately leading to a poorer prognosis [143].

### 4.2. Respiratory System

#### 4.2.1. Lung Adenocarcinoma

Lung adenocarcinoma (LUAD) represents the most prevalent histological subtype of primary lung cancer that has high morbidity and mortality rates worldwide [144]. M6A methylation of specific genes has been identified in the development of LUAD, and studying genes associated with different m6A modification patterns in TME could enhance early tumor diagnosis and reveal new immunotherapeutic targets [145,146].

Recent studies suggest that TME immune cells may play an oncogenic role in influencing immunotherapy through the upregulation of M6A-associated regulators. The expression of leucine-rich pentatricopeptide repeat-containing protein (LRPPRC), a reader of m6A, was found by Ji Ke et al. to be upregulated in tumor tissues. It was negatively correlated with the degree of immune cell infiltration, especially dendritic cells, CD8+ cytotoxic T lymphocytes, and macrophages. Moreover, patients with high LRPPRC expression had a low response rate to PD-1 immunotherapy. Thus, it was hypothesized that LRPPRC expression may affect the response to immunotherapy by inhibiting the infiltration and activation of immune cells in the tumor microenvironment, and acts as a potential immunotherapy marker [77]. Similarly, Lige Wu et al. demonstrated that M2-type tumor-associated macrophages (M2-TAMs) may attenuate the antitumor immune response of CD8+ T cells in lung adenocarcinoma (LUAD) through the upregulation of METTL3 in tumor cells. Consequently, compared to patients with LUAD who are responsive to nivolumab, those resistant to the therapy exhibit higher levels of M2-TAM infiltration and reduced infiltration of CD8+ T cells and natural killer (NK) cells in the primary tumor tissues [78].

Wang et al. reported that sustained antigen exposure stimulates the expression of lymphocyte-activation gene 3 (LAG3), a pivotal immune checkpoint regulator, on CD4+ and CD8+ T cells. This induction leads to the functional suppression and eventual exhaustion of these cells [79]. Elevated LAG3 expression strongly correlates with the infiltration levels of CD4 memory activated T cells, resting NK cells, M0 macrophages, M1 macrophages, and mast cells in the tumor microenvironment. Intriguingly, inhibition of LAG3 within this milieu is associated with poorer prognosis [80].

Additionally, m6A-modified long non-coding RNAs (lncRNAs) are strongly associated with immune cell infiltration, particularly gamma delta T cells and neutrophils, as well as with tumor mutational burden. Risk models based on m6A-related lncRNAs demonstrates a superior predictive capability [147,148,149].

#### 4.2.2. Nasopharyngeal Carcinoma

Nasopharyngeal carcinoma (NPC), originating from squamous epithelial cells of the nasopharyngeal mucosa, represents the most prevalent malignant tumor in the head and neck region [150,151]. LRPPRC, which acts as m6A ‘reader’, in NPC exhibits a negative correlation with Th2 cell infiltration but a positive correlation with protective immune cells such as Tcm, B, and T cells. It plays an important role in immune cell activity and the transition from epithelial to malignant cells [81].

ALKBH5 serves as an independent risk factor for overall survival in HNSCC [152]. In HNSCC patients, upregulated ALKBH5 is inversely associated with RIG-I and IFNα expression. ChIRP-MS analysis revealed that HNRNPC binds to the m6A sites of DDX58 mRNA, facilitating its maturation. Overexpression of ALKBH5 suppresses retinoic acid-inducible gene I (RIG-I)-mediated IFNα secretion via the IKKε/TBK1/IRF3 pathway [82]. Another study also demonstrated that in immunocompetent C3H mice, ALKBH5 overexpression reduces tumor-infiltrating lymphocytes, which can be restored by IFNα treatment [153].

### 4.3. Endocrine System

#### 4.3.1. Thyroid Cancer

Thyroid cancer stands out as one of the most prevalent malignancies within the endocrine system [154]. However, treatment options for advanced papillary thyroid cancer (PTC) and anaplastic thyroid cancer (ATC) that are refractory to standard therapies remain limited and unstable [155]. Ning et al. demonstrated a negative correlation between METTL3 expression and CD70 expression, as well as infiltration of M2 macrophages and regulatory T cells (Tregs) in PTC and ATC tissues. Moreover, the stabilization of CD70 mRNA and subsequent upregulation of CD70 protein levels increased the presence of immunosuppressive Tregs and terminally exhausted T cells, leading to resistance against anti-PD-1 therapy. The blockade of CD70 using cusatuzumab, a high-affinity monoclonal antibody, reversed the resistance to anti-PD-1 therapy induced by M2 extracellular vesicle (EV) in vivo [83]. In contrast, YTHDF3 expression exhibited a positive correlation with the infiltration level of CD4+ T cells and macrophages, and it was closely linked with various immune markers [156]. These findings offer a new perspective on comprehending the modulation of m6A RNA in TC and its relevant immunotherapy strategies.

#### 4.3.2. Pituitary Adenomas

Pituitary adenomas (PAs) encompass neoplasms originating from the pituitary adenohypophyseal cell lineage, including functioning tumors that secrete pituitary hormones, and nonfunctioning tumors [157]. YTHDF2 exhibited high expression levels in PAs, concomitant with increased M2 macrophages and PD-L1 expression, positioning it as a promising biomarker for immunotherapy and a potential molecular target in PAs [84]. METTL3 and methylated RNA may constitute suitable targets for GH-PA clinical therapy, but the relevant immune mechanisms remain unclear [158].

### 4.4. Nervous System

Gliomas are the most commonly occurring primary and potentially fatal intracranial tumors [87]. About half of glioma cases are diagnosed as glioblastoma (GBM), known for its aggressive nature, presents a challenge due to the limited availability of effective therapeutic options [159]. Since GBM shows satisfied response to immune therapy, m6A regulators that correlated with PD1 or cytokines may act as therapeutic targets for GBM. Recent studies have indicated that m6A methylation is important for immune cell infiltration, maintenance of cell renewal, and therapy resistance in GBM.

HSPA7 was found to promote macrophage infiltration and SPP1 expression by upregulating YAP1 and LOX in glioblastoma stem cells (GSCs), both in vitro and in clinical GBM tumor samples. Additionally, silencing HSPA7 showed promise in enhancing the efficacy of anti-PD1 therapy in a GBM model, highlighting its potential as a novel target for immunotherapy [88].

ALKBH5 was upregulated in GBM, and higher ALKBH5 expression was associated with a poorer prognosis [160]. ALKBH5 deficiency improved the immune microenvironment by increasing the number of CD4+ and CD8+ T lymphocytes and enhancing the ratio of CD8+/CD4+ T cells. Tang et al. also observed that knocking out ALKBH5 activated the immune system, leading to increased antitumor-associated cytokines (IFN-γ, IL-2) and reduced pro-tumor-associated cytokines (IL-10, IL-13), reducing PD-L1 protein levels both in vitro and in vivo [86].

In exploring other m6A related immunological roles of GBM, Lin et al. revealed that FTO, ZC3H13, and YTHDC1 positively correlated with T cells, while macrophages showed a negative correlation with FTO and ZC3H13 [85]. Moreover, WTAP and HNRNPC was found to be overexpressed and regulated migration and invasion in vitro. These could serve as a potential prognostic biomarker and therapeutic target for GBM [161].

Some reports suggest that ferroptosis is associated with the tumor microenvironment and posttranscriptional regulation [162,163]. In lower-grade glioma (LGG), elevated expression of transferrin receptor protein 1 (TFRC) was linked to an increase in immune cells, including CD8+ T cells, macrophages, and myeloid dendritic cells. Furthermore, there was a strong correlation between TFRC expression and certain immune checkpoint-related molecules, such as PD-L1 (CD274) and CTLA4 [164].

### 4.5. Urinary System

#### 4.5.1. Prostate Cancer

Prostate cancer (PCa) represents a significant threat to men globally [94,165]. While radical surgery and radiation therapy remain effective treatments for early-stage PCa, late-stage diagnoses due to limited screening awareness necessitate endocrine therapy as a crucial approach [95,96]. Moreover, methylation modification and immune cell infiltration play a non-negligible role in prostate cancer for personalized immune therapy. However, because PCa shows bad response to immune therapy, the m6A regulator for the TME of PCa is still in the process of laboratory trail.

Recent studies have highlighted the relevance of m6A regulators in PCa, linking their expression to genetic variation, alternative splicing (AS), tumor mutation burden (TMB), and TME [166]. Liu et al. observed that elevated levels of METTL14 and ZC3H13 were associated with increased Th1 and Th17 cells, stromal fraction, and TGF-beta response. Th1 cells are known for triggering antitumor immune responses, while Th17 cells serve as a favorable prognostic marker and have been linked to the efficacy of PD-1 blockade therapy in PCa [39,89]. In addition, alterations in METTL3 expression were found to negatively correlate with regulatory T cells and M2 macrophages in PCa, impacting treatment outcomes significantly [167].

The proportion of T cells CD8 is strongly correlated with the set of immune-related genes. A study demonstrated that HNRNPC inhibits PCa tumor immunity by boosting Treg cell activation and suppressing effector CD8 T cells [90]. Therefore, targeting HNRNPC to activate the immune microenvironment may offer a potential therapeutic option for advanced PCa [168].

#### 4.5.2. Renal Cell Carcinoma

Renal cell carcinoma (RCC) is the most common urogenital cancer, with clear cell renal cell carcinoma (ccRCC), papillary renal cell carcinoma (pRCC), and chromophobe renal cell carcinoma (chRCC) being the most prevalent types [169]. The functions of m6A modulators vary across different RCC types. Jiang et al. discovered that the expression of METTL14 in kidney renal clear cell carcinoma (KIRC) is correlated with immune cell infiltration levels, traditional immunological checkpoints (e.g., CD274, HAVCR2), and potentially associated with the NF-κB pathway [91,92,93]. Another study revealed that the expression level of METTL5 in human KIRC and papillary RCC (KIRP) tissues was notably higher than in their respective normal neighboring tissues. It exhibited a negative correlation with the infiltration level of NKT cells and naïve CD8+ T cells, and a positive correlation with the infiltration ratio of memory CD4+ T cells, T-helper 2 (Th2) CD4+ T cells, (non-regulatory) CD4+ T cells, and ordinary lymphoid progenitor cells [97].

In pRCC, evaluating the antitumor immune effects within TME can be accomplished by examining T cell markers such as CD8 and CD69, as well as macrophage markers such as CD163. Additionally, analyzing the differential expression of m6A regulators such as YTHDF1 and METTL14 can contribute to validating the prognostic significance of immunotherapy in clinical contexts [170].

The expression level of CDH13 in ccRCC showed a significant correlation with the expression levels of many m6A-regulated effector proteins and was strongly linked to the enrichment of CD56bright NK cells, mast cells, NK cells, and pDC [171]. Moreover, ALDOB is also strongly regulated by m6A-regulated effector proteins and influences immune infiltration in ccRCC [98].

#### 4.5.3. Bladder Cancer

Bladder urothelial carcinoma (BLCA) is a malignancy associated with high morbidity and mortality rates [172]. It typically originates in the bladder mucosa and stands as the most frequently diagnosed malignancy in the genitourinary system [173,174,175,176]. Studies have shown that ENO1 and PGM1 contribute to cancer progression by influencing the glycogen synthesis pathway [99]. This process is significantly positively correlated with FTO and IGF2BP3 expression, while exhibiting a notable negative correlation with YTHDC1 [177,178]. Zhao et al. suggested that PGM1 and ENO1 were significantly positively associated with the M2 surface marker CD163 but negatively correlated with the TFH cell marker CXCR5. Conversely, YTHDC1 was negatively associated with M2 macrophage infiltration and positively correlated with TFH cell infiltration [99]. These findings shed light on the relationship between the expression of m6A methylation regulators in BLCA and immune cell infiltration.

### 4.6. Reproductive System

#### 4.6.1. Breast Cancer

Breast cancer (BC) stands out as the most lethal malignancy among women [179,180]. YTHDF1 has been proved to promote breast cancer cell growth, DNA damage repair, and chemotherapy resistance [181]. Furthermore, YTHDF1 expression exhibited notable correlations with activated CD4 memory T cells, M1 macrophages, activated NK cells, and monocytes in breast cancer tissues. These associations might also coincide with a higher tumor mutational burden (TMB) score, suggesting that patients with elevated YTHDF1 levels could potentially derive greater benefits from immunotherapy [109].

Discoidin domain receptor 1 (DDR1), a non-integrin collagen tyrosine kinase receptor, was found to be overexpressed in BC and inversely associated with the proportion of infiltrating antitumor immune cells [182,183]. According to Lian et al., HNRNPC facilitated the expression of TFAP2A and subsequently the transcription of DDR1 by recognizing the m6A modification of TFAP2A mRNA mediated by VIRMA—these mainly through potentiating the TFAP2A/DDR1 axis and promoting collagen fiber alignment, thus decreasing the infiltration of antitumor immune cells and fostering immune evasion in BC [110].

#### 4.6.2. Cervical Cancer

Cervical cancer ranks as the fourth most prevalent cancer among women globally [184], with 604,000 new cases and 342,000 deaths reported in the Global Cancer Statistics 2020 [172]. The expression of PD-L1 significantly increases in cervical cancer tissues and demonstrates a notable correlation with the expression levels of ALKBH5, FTO, METTL3, RBM15B, YTHDF1, YTHDF3, and ZC3H13 [100].

IGF2BP3 exhibits high expression in human cervical cancer and plays a crucial role in stabilizing the mRNA of GLS and GLUD1 genes, which are key metabolic enzymes in glutamate and glutamine metabolism, through m6A modification [101,102]. This process leads to immune escape in cervical cancer by promoting lactate production and secretion [185].

METTL3 demonstrated a correlation with cell migration, chemotaxis, and cytokines, all playing pivotal roles in the immune response [186]. The direct induction of CD33+CD11b+HLA-DR-myeloid-derived suppressor cells (MDSCs) and tumor-derived MDSC induction in vitro were observed to be attenuated in the absence of METTL3 [103]. In cervical squamous cell carcinoma (CESC), ICOS, KIR2DL4, TNFSF9, and CD86 function in immune activation and display a negative association with METTL3 expression. Conversely, other immune-activating molecules such as BTN2A2, VTCN1, PD-L1, CD47, BTNL9, PVR, and TNFRSF14 exhibit a positive correlation with METTL3 expression levels in HNSC [83,187]. As a result, according to the study, the expression of PD-L1 in CSCC exhibits considerable variation, ranging from 19% to 88% and thus suggesting a complex and potential treatment [188].

#### 4.6.3. Endometrial Cancer

Globally, endometrial cancer (EC) ranks as the second most frequent gynecological malignancy, following only cervical cancer [189]. TIMER algorithm shows that immune cell infiltration is associated with changes in ZC3H13, YTHDC1, and METTL14 expression [104]. METTL14 showed a positive correlation with CD4+ T cell infiltration and a negative correlation with macrophage infiltration [105,106]. Additionally, ZC3H13 and YTHDC1 expression were positively correlated with CD4+ T cell infiltration but negatively correlated with macrophage and NK cell infiltration. Notably, the expression levels of these enzymes were positively associated with PD-L1 expression in EC, suggesting that these proteins may enhance the outcome of immunotherapy [104,190,191,192].

#### 4.6.4. Ovarian Cancer

Ovarian cancer (OC) ranks as the eighth most commonly diagnosed cancer and the eighth leading cause of cancer-related deaths among women [193]. In 2020, it is estimated that there were approximately 314,000 new cases of ovarian cancer worldwide, accounting for 3.4% of all cancer cases, and approximately 207,000 deaths, representing 4.7% of all cancer deaths globally [194,195]. In recent years, although immune checkpoint inhibitor (ICI) therapy has been used in many solid tumors, it seems that the inhibitory effect of TME in OC reduces the efficacy of ICI therapy. However, m6A regulators, which can influence the TME function, may contribute to improving the effectiveness of immune therapy for OC.

CircNFIX is an oncogene in OC that promotes malignant proliferation, metastasis, and angiogenesis. Wang Rui et al. proved that M6A-modified circNFIX promotes ovarian cancer progression and immune escape by activating IL-6R/JAK1/STAT3 signaling by sponging miR-647 [196]. Additionally, the expression of CDC42EP3 correlates with various tumor-infiltrating immune cells, including NK cells, Tcm cells, and Tgd cells [107,108]. CDC42EP3 may contribute to modulating a wide range of immunoregulatory molecules in ovarian cancer, potentially influencing the infiltration and functions of immune cells within TME [197]. Through bioinformatics analysis, pan-cancer studies, and experimental validation, numerous other m6A-related target genes, lncRNAs, and regulatory proteins have been identified, shedding light on their roles in modulating OC immunotherapy and prognosis [198,199,200].

### 4.7. Musculoskeletal System

#### 4.7.1. Melanoma

Melanoma ranks among the most lethal and therapy-refractory cancers in humans, presenting significant challenges in treatment and survival rates [201]. FTO plays a crucial role as an m6A demethylase in driving the melanomagenesis, immunoinflammatory phenotype. This suggests that combining FTO inhibition with anti-PD-1 blockade may potentially overcome resistance to immunotherapy in melanoma [111,112].

In a study by Li et al., ALKBH5 was found to be essential for regulating in vivo tumor growth and regulating lactate and Vegfα accumulation in TME during GVAX/anti-PD-1 treatment. Mct4/Slc16a3 was identified as a key target of ALKBH5 in this process [138]. ALKBH5-KO B16 tumors exhibited reduced expression of Mct4/Slc16a3, lactate content in the tumor-infiltrating fluid (TIF), and decreased abundance of myeloid-derived suppressor cells (MDSCs) and regulatory T cells (Tregs) within TME [113,114].

#### 4.7.2. Osteosarcoma

Osteosarcoma (OS) is the most prevalent primary bone malignancy, predominantly affecting children and adolescents [202,203]. Long non-coding RNAs (lncRNAs) play a crucial role in determining the prognostic value and immune landscapes of OS [204]. Zheng et al. emphasized AC004812.2 as a protective factor in OS, with low expression indicating poorer overall survival. Overexpression of AC004812.2 was found to suppress the proliferation of 143B cells while increasing the expression levels of IGF2BP1 and YTHDF1 [205]. Furthermore, DEPTOR expression showed a negative correlation with NBR2 expression and a positive correlation with AC036214.2 and AL161785.1, which were closely associated with the infiltration of immune plasma cells [206].

### 4.8. Acute Myeloid Leukaemia

Acute myeloid leukemia (AML) is a prevalent hematopoietic malignancy characterized by the hyperplasia of myeloid blood cells, resulting in the rapid growth of abnormal cells in the bone marrow and blood, disrupting hematopoietic function. AML patients often face poor prognoses and low overall survival (OS) rates due to the disease’s heterogeneity and the complex nature of its TME [207,208]. Currently, M6A-associated lncRNAs assessed using risk prediction models have the potential to predict prognosis and design immunotherapies in AML patients [209,210]. According to Liao et al., the majority of immune cells exhibited a negative correlation with METTL14 expression and a positive correlation with ZC3H13. Additionally, CTLA-4 demonstrated a negative correlation with both METTL14 and ZC3H13. On the other hand, PD-L1 displayed a positive relationship with YTHDC2 but a negative correlation with RBM15 [115]. These lncRNAs may target common genes such as FTO and MYC, leading to similar biological consequences through distinct mechanisms [211].

## 5. m6A Modification-Association with Immune Regulation and Implications on Autoimmune and Immune-Related Diseases

**Figure 3:**
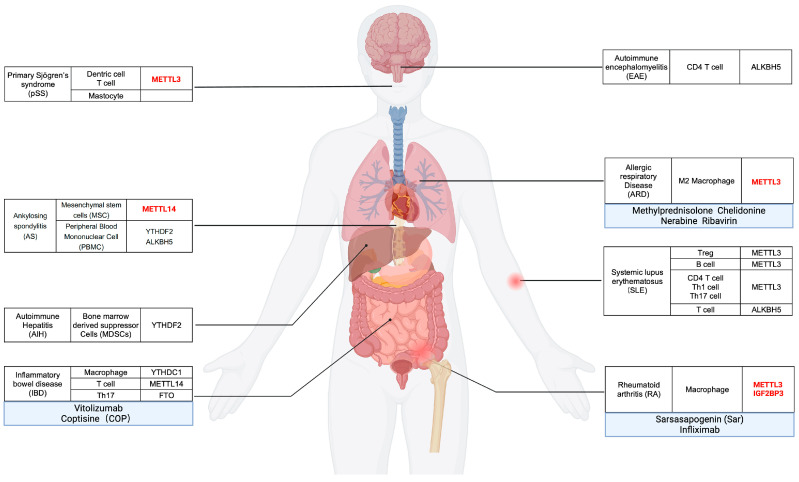
Immunity-related m6A modifiers promote immune-related diseases. Immune cells can be regulated by modifiers (the positively regulated (upregulated) are in red, while the negatively regulated (downregulated) are in black) to cause immune-related diseases. M6A-specific immune-related therapy is shown in blue background.

Autoimmune diseases occur when the immune system malfunctions, attacking the body’s own tissues. The precise triggers of these disorders remain unclear, but they are generally attributed to a confluence of genetic, environmental, infectious, and psychological factors, leading to the emergence of autoantigens, abnormal immune regulation, and genetic predispositions [212]. Traditional management of autoimmune diseases typically involves immunosuppressive therapies, which, while effective, can lead to significant side effects, including increased susceptibility to severe infections. Consequently, there is a growing interest in identifying novel therapeutic targets and strategies. Emerging evidence underscores the pivotal role of N6-methyladenosine (m6A) in regulating immune cell functions, thereby influencing the pathogenesis of autoimmune diseases. This suggests that m6A may represent a promising target for epigenetic therapy in these conditions (Figure 3) (Table 2).

### 5.1. Rheumatoid Arthritis, RA

Rheumatoid arthritis (RA) is a prevalent chronic autoimmune condition characterized by disrupted immune tolerance [234]. The disease is mediated by complex interactions among various immune cells, including dendritic cells, T cells, macrophages, B cells, neutrophils, fibroblasts, and osteoblasts. A critical pathological feature of RA is the hyperproliferation of synovial fibroblasts. N6-methyladenosine (m6A) methylation plays a crucial role in the interactions between RA-related immune cells and the inflammatory cytokines TNF-α and IL-1β, which are secreted by fibroblast-like synoviocytes (FLSs) located on the synovial membranes at the initial sites of the disease [235].

Comprehensive analysis of RNA-seq and single-cell RNA-seq datasets has identified IGF2BP3 as a key regulator in the proliferation of RA-FLS by influencing the G2/M phase transition and promoting M1 macrophage polarization, which exacerbates inflammation [213]. Furthermore, research by Lei et al. demonstrated a reduction in PTEN mRNA methylation in RA synovial tissues, coinciding with an abnormal activation of the PI3K-Akt signaling pathway that promotes the proliferation of synovial fibroblasts [236,237]. These mechanisms contribute to symptoms such as synovial swelling, pain, and functional limitations during the acute phase of RA.

METTL3, the primary enzyme responsible for m6A methylation modification, plays a crucial role in immune and inflammatory regulation in rheumatoid arthritis (RA). Studies have shown that METTL3 expression is elevated in peripheral blood monocytes and fibroblast-like synoviocytes (FLSs) compared to controls. This upregulation enhances dendritic cell-mediated T cell responses and exerts inhibitory effects on LPS-induced inflammation through the NF-κB signaling pathway [15,214]. Notably, a cross-validated study revealed that Sarsasapogenin (Sar) could serve as a novel anti-RA drug by suppressing the expression of key regulators involved in DNA replication, cell cycle, and apoptosis, thereby impairing the NF-κB signaling pathway and ameliorating RA progression [238]. Additionally, METTL3 has been observed to promote M1 macrophage polarization through direct methylation of STAT1 mRNA, highlighting a potential target for anti-inflammatory therapy in RA [41].

Further investigations into RA pathogenesis and risk factors have identified altered expression patterns of m6A-related modifiers in peripheral blood and synovial tissues. Specifically, decreased levels of ALKBH5, FTO, and YTHDF2 were observed in peripheral blood, while in synovial tissues, METTL3, METTL14, WTAP, METTL16, and YTHDF2 were significantly upregulated, and FTO was markedly downregulated. Potential target genes implicated in these changes include CHD3, SETD1B, FBXL19, SMARCA4, and SETD1A, all associated with immune cell function and inflammatory responses [239,240,241]. Additionally, Shan Song et al. developed a scoring model demonstrating the clinical utility of the m6A score as a biomarker for predicting response to infliximab therapy in RA. In clinical practice, Rituximab is primarily used to mitigate RA symptoms by suppressing B cell function, particularly when TNF inhibitors fail, with therapeutic outcomes varying based on the m6A modification profile [242]. These insights undeniably offer new perspectives for the diagnosis and therapeutic strategies in RA.

### 5.2. Systemic Lupus Erythematous, SLE

Systemic lupus erythematosus (SLE) is a chronic systemic autoimmune disorder characterized by the formation of immune complexes due to abundant autoantibodies, leading to inflammation and organ tissue damage [243]. Several studies have demonstrated that the mRNA levels of METTL3, METTL14, WTAP, FTO, ALKBH5 and YTHDF2 are significantly reduced in the peripheral blood of SLE patients compared to healthy controls, highlighting the crucial role of m6A modifications in the disease’s pathogenesis [244,245].

A defining feature of SLE, the production of anti-double-stranded DNA (anti-dsDNA) antibodies, correlates negatively with ALKBH5 mRNA levels, suggesting a potential mechanism in the disease’s development [244]. Additionally, METTL3 enhances the stability of FOXO3 mRNA through m6A modification of its 3′-untranslated region, facilitated by YTHDF1 interaction [246]. In patients with decreased anti-dsDNA antibody levels, a reduction in B cell-triggered FOXO3 exacerbates disease progression [217].

Abnormal T cell subset distribution and excessive CD4+ T cell activation are central to SLE pathogenesis [247]. Recent findings suggest that m6A modifications are vital for maintaining T cell homeostasis. Specifically, decreased METTL3 levels in SLE CD4+ T cells lead to overactivation and disrupt the balance of effector T cell differentiation, contributing to elevated Th1 and Th17 subsets and reduced regulatory T (Treg) cell differentiation in mouse models [218]. Moreover, in Mettl3^–/–^Tregs, elevated levels of SOCS proteins obstruct signal transduction from the IL2-STAT5 pathway, which is critical for Treg function and homeostasis maintenance [215,216]. Besides, Deng et al. reported reduced ALKBH5 expression in both PBMCs and T cells of SLE patients, which inhibits apoptosis and promotes T cell proliferation [248]. Collectively, these findings underscore the significance of m6A modification in regulating T cell differentiation and development, and its essential role in the progression of SLE, providing new avenues for understanding and potentially treating this complex autoimmune condition.

The autoimmune response in systemic lupus erythematosus (SLE) often leads to inflammation and subsequent damage in various organs, such as the joints, kidneys, and skin. Specifically, lupus nephritis (LN) is characterized by increased interstitial fibrosis. Research indicates that genistein, a soy-derived isoflavone, can enhance ALKBH5 expression in the kidneys during unilateral ureteral obstruction (UUO)-induced fibrotic kidney disease, subsequently decreasing RNA m6A methylation levels and ameliorating renal damage [219,220]. Additionally, m6A modifications mediated by METTL3 have been implicated in the dysfunction of bone marrow-derived mesenchymal stem cells (BM-MSCs), which may contribute to osteoporosis through activation of the NF-κB pathway and increased IFN-β accumulation [249,250,251]. These outcomes show that RNA methylation also serves as major contributor to organ damage.

### 5.3. Inflammatory Bowel Disease, IBE

Inflammatory bowel diseases (IBDs), comprising Crohn’s disease (CD) and ulcerative colitis (UC), represent idiopathic chronic inflammatory disorders of the intestinal mucosa. The etiology and pathogenesis of these conditions involve genetic predispositions, intestinal microbiota, and host immune responses, yet remain incompletely understood. Comprehensive analyses of extensive microarray and RNA-seq datasets across multiple IBD studies have elucidated a landscape of m6A modifications, establishing a significant association between m6A-related genes and regulatory factors in IBD pathogenesis [252].

The integrity of the intestinal mucosa’s immune system is maintained by a robust barrier composed of closely packed intestinal epithelial cells (IECs), intraepithelial lymphocytes, M cells, and other immune components, serving as the primary defense against pathogenic invasions. Disruptions in this barrier are pivotal in the pathophysiology of IBD, characterized by chronic intestinal inflammation. m6A modification plays a critical role in regulating apoptosis and autophagy within intestinal epithelial cells and in modulating mucosal immunity. Notably, METTL14 has been shown to maintain the homeostasis of colonic epithelial cells by inhibiting the NF-κB-mediated anti-apoptotic pathway and by stabilizing Nfkbia mRNA [253]. Additionally, research by Ge et al. identified that YTHDC1 in macrophages fine-tunes the expression of NME nucleoside diphosphate kinase 1 (NME1), thereby enhancing the integrity of the colonic epithelial barrier, further highlighting the intricate regulatory roles of m6A modifications in IBD [221].

The pathogenesis of colitis is intricately linked to dysfunctional Treg cells and influenced by the microbiome. Studies investigating colitis pathogenesis have revealed that Mettl14-deficient Treg cells fail to adequately suppress inflammation induced by naïve T cells in a colitis model, demonstrating that the absence of METTL14 in T cells can lead to spontaneous colitis [254]. Additionally, in an IL-10^–/–^mice with Th1 response, CD4-Cre^+/Tg^ Mettl14^FL/FL^ conditional knockout mice consistently develop colitis by week six [254,255]. On the other hand, the gut microbiota plays a role in modulating m6A modification by regulating protein expression. For instance, Escherichia coli triggers an increase in overall m6A levels, promoting enteric defensin expression [256]. In intestinal epithelial cells exposed to LPS, METTL3 overexpression exacerbates cellular inflammation in mice [257]. Researchers have also observed a significant decrease in m6A modifications in colorectal cancer (CRC) cells due to the downregulation of the m6A methyltransferase METTL3 induced by Fusobacterium nucleatum (F. nucleatum), contributing to CRC aggressiveness. Conversely, alterations in host m6A modification can impact gut microbiota composition, suggesting innovative approaches for developing microbiome-targeted therapies for IBDs [254,258].

The insights gained from the research on IBD development underscore the significant potential of m6A-related immunological modifications for clinical applications in understanding and managing IBD. M6A regulators and phenotype-related hub genes could play a crucial role in developing innovative therapeutic options. Currently, the predominant treatments revolve around biological agents such as anti-TNF (primarily infliximab) [259]. Chen et al. observed that IBD patients with high expression of certain m6A phenotype-related hub genes (H2AFZ, NUP37, SNRPD1, CPSF3, and RBBP7), along with elevated infiltration levels of M1 macrophages, M0 macrophages, naïve B cells, CD4 memory-activated T cells, memory B cells, and activated DCs, might exhibit resistance to infliximab [260]. Furthermore, the presence of integrin α4β7 in Th17 cells within the lamina propria tissue of UC patients with reduced FTO expression could potentially enhance their response to vedolizumab treatment [223]. Additionally, compounds such as coptisine (COP) show promise in countering DSS-induced UC by upregulating METTL14 expression, thereby enhancing m6A methylation and exerting an anti-inflammatory effect on macrophages [222]. Recent studies have been also exploring m6A-related targets for adjuvant chemotherapy and direct therapy. Further investigations are necessary to optimize therapeutic strategies for IBD patients, reflecting ongoing efforts to leverage m6A-related mechanisms for improved clinical outcomes [261,262,263].

### 5.4. Primary Sjogren Syndrome, pSS

Primary Sjögren’s syndrome (pSS) is a prevalent autoimmune disorder characterized by lymphocyte infiltration into exocrine glands, particularly the lacrimal and salivary glands, resulting in reduced tear and saliva production [264]. M6A modification may influence the development of pSS by regulating gene expression and immune cell responses. The mRNA levels of m6A regulators in PBMCs (such as FTO, YTHDF2, YTHDF3, or YTHDC2) might enhance ISG15 expression, thereby activating the type I interferon (IFN) signaling pathway and contributing to autoimmunity initiation in primary pSS [265]. Furthermore, FMR1 emerges as a pivotal gene in distinct m6A modification patterns observed in pSS blood and parotid samples. Variations in immune cell infiltration were noted between high and low FMR1 expression groups (blood) or HNRNP/FMR1 expression groups (parotid gland), supporting FMR1’s role as an immunomodulator in pSS. The negative correlation between FMR1 and mast cells, implicated in tissue fibrosis during pSS development, suggests FMR1’s involvement in inflammatory fibrosis remission in parotid tissue. Additionally, the interplay between upregulated m6A-related autophagy pathways and immune infiltration contributes to pSS pathogenesis. Notably, pSS exhibits heterogeneity, with a pronounced B cell infiltrate associated with elevated anti-Ro/SSA antibody levels. These findings shed light on the complex immunological mechanisms underlying pSS and highlight potential therapeutic targets for managing this condition [225].

Furthermore, a correlation analysis confirmed a comprehensive linkage between ALKBH5 and METTL3 with infiltrating immune cells. Notably, METTL3 exhibited the strongest negative correlation with the abundance of activated DCs and plasmacytoid DCs in pSS. These findings are consistent with the notion that mRNA methylation, mediated by METTL3, promotes DC activation and T cell stimulation in pSS [224].

### 5.5. Ankylosing Spondylitis, AS

Currently, it is widely accepted that immune system dysregulation drives the onset and progression of ankylosing spondylitis (AS), characterized by chronic inflammation and ectopic ossification in the entheses [266]. In terms of pathogenesis, mesenchymal stem cells from AS patients (AS-MSCs) exhibit enhanced osteogenic differentiation compared to normal subjects. This enhanced differentiation is attributed to the less consistent long-chain non-coding RNAs and mRNAs associated with osteogenic differentiation in AS-MSCs compared to normal MSCs. Specifically, CCL2 secretion by AS-MSCs during enhanced osteogenic differentiation increases the polarization of pro-inflammatory macrophages and enhances TNF-α secretion at the attachment sites by promoting monocyte migration. Furthermore, TNF-α-mediated METTL14-dependent m6A modification of ELMO1 triggers directional migration of MSCs in ankylosing spondylitis, leading to pathological osteogenesis and ligamentous osteoid formation [267]. Additionally, the ALKBH5–PRMT6 axis controls the activation of the AKT signaling pathway to modulate the osteogenesis of MSCs [226]. Future studies may focus on developing small molecule inhibitors targeting ALKBH5 and ELMO1 to improve the clinical outcomes of MSC transplantation for the treatment of low bone mass-related diseases.

In human AS peripheral blood mononuclear cells (PBMCs), the mRNA levels of YTHDF2 and ALKBH5 were notably lower compared to those in healthy controls. Notably, a combination of mRNA YTHDF2 and SII serves as an indicator of disease activity and severity [227].

Bioinformatics technology proves significant in elucidating the potential predictive and regulatory mechanisms of m6A regulatory factors in AS [268]. Through a novel predictive model utilizing unsupervised machine learning (UML) clusters built using three predictive factors—C-reactive protein (CRP), absolute value of neutrophils (NEU), and absolute value of monocytes (MONO)—Sun et al. discovered a novel subtype of AS and a correlation between FTO expression and immune cell infiltration. The three m6A characteristic genes, RBMX, METTL14, and ALKBH5, show a negative relation to AS, and immune correlation analysis revealed their association with immunity, particularly RBMX, suggesting their significance in AS [269].

### 5.6. RNA Methylation and Other Autoimmune Diseases

Many studies have confirmed the interplay between RNA methylation modifications and the biology of immune cells in various autoimmune diseases. In autoimmune hepatitis (AIH), upregulation of YTHDF2 decreases the expansion, chemotaxis, and suppressive function of bone-marrow-derived suppressor cells (MDSCs), offering a distinctive therapeutic target for immune-mediated hepatitis [228].

In autoimmune encephalomyelitis (EAE), ALKBH5 deficiency increases m6A modification on IFNG and CXCL2, suppressing the IL-17 signaling pathway in CD4+ T cells during EAE and attenuating CD4 T cell responses, as well as neutrophil recruitment into the central nervous system [229]. For allergic respiratory disease (ARD), METTL3 participates in the Th1/Th2 imbalance and partially inhibits M2 macrophage activation via the PI3K/AKT and JAK/STAT6 signaling pathways [230]. In liver fibrosis and nephritis, m6A has been identified as a mediator of inflammation [270,271,272].

The crosstalk between m6A modification and non-coding RNAs presents peculiarities and could guide future exploration [273]. The long non-coding RNA (lncRNA) UCA1 can enhance keratinocyte-driven inflammation by inhibiting METTL14 and activating the HIF-1α/NF-κB pathway in psoriasis [274]. Similarly, hsa_circ_0004287 inhibits M1 macrophage activation in an m6A-dependent manner in psoriasis, suggesting its potential as a therapeutic candidate [275].

### 5.7. M6A and Acquired Immunodeficiency Syndrome, AIDS

Emerging evidence indicates that m6A modification may play a crucial role in the viral lifecycle and infection process. Human immunodeficiency virus type 1 (HIV-1), a member of the Lentivirus genus within the Retroviridae family, is responsible for causing acquired immunodeficiency syndrome (AIDS) by mainly targeting the body’s immune system, including CD4+ T lymphocytes, macrophages, and dendritic cells.

Three studies in 2016 revealed that HIV-1 genomic RNA is decorated with m6A at 5′UTRs, env/rev, and 3′UTRs, confirming the regulatory effect of m6A in the lifecycle of certain viruses [231,232,233]. Lichinichi et al. observed an increased prevalence of the MGACK (A/C-GAC-G/U) motif in infected T cells. Conversely, Tirumuru et al. noted a slight rise in RRACH motif enrichment and a reduction in the GGACU motif in infected primary CD4+ T cells. Using meRIP-seq and PA-m6A-seq techniques, seven common sites were identified across all three studies to reveal the modification state of fragmented RNA.

Interestingly, two studies using different mapping strategies found the presence of m6A at specific additional sites (such as RRE) within the viral genome [231,232,233].

Studies have suggested that downregulation of METTL3 and METTL14 leads to decreased levels of CAp24 and/or Gag proteins in infectious immune cells and supernatants, as well as total levels of gp120 mRNA. Conversely, downregulation of FTO and/or ALKBH5 has the opposite effects. However, the function of cytoplasmic m6A readers of the YTHDF family, particularly YTHDF1, remains ambiguous. Kennedy et al. and Lichinchi et al. demonstrated that all three YTHDF proteins bind to HIV RNA and promote HIV replication. In contrast, findings from Tirumuru et al. suggested that all three YTHDF proteins antagonize HIV replication. These discrepancies are likely due to variations in cell types, reagents, and experimental methods used in the studies [276].

In contemporary research, advanced tools such as CRISPR/Cas9 technology are employed to delve deeper into molecular mechanisms, including the m6A modification of HIV-1 RNA and its impact on various stages of the viral lifecycle. These insights into m6A modification offer potential avenues for developing novel therapeutic strategies to combat viral infections.

## 6. Conclusions and Prospects

In recent years, there has been a notable increase in studies investigating the biological role of m6A methylation in diverse immune cell populations. Initially, research efforts predominantly focused on understanding the physiological functions of m6A modifications in immune cells involved in both innate and adaptive immune responses. As our understanding of these normal functions has expanded, there has been a growing emphasis on exploring the pathological implications of m6A modification in various systemic diseases [277,278,279]. It is important to recognize that the mechanisms through which m6A influences different immune and tumor cell types remain incompletely understood due to the dynamic and intricate nature of m6A regulation, coupled with the ongoing discovery of new regulatory factors. Consequently, fully deciphering how m6A modulators govern immune cell behavior presents a challenge, yet it holds the potential to advance our comprehension of m6A’s biological significance in disease-related immunity and to furnish robust evidence supporting the development of targeted cancer immunotherapies.

**Figure 4:**
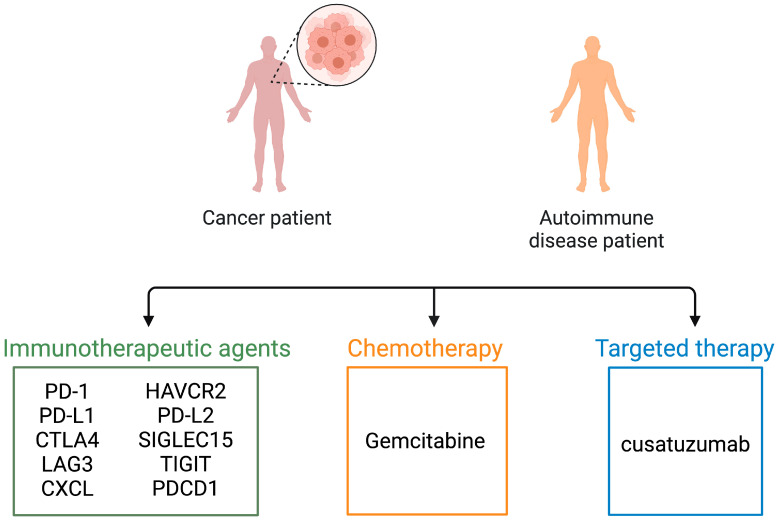
m6A modification as a biomarker for potential therapies in oncology and autoimmune disease. m6A modification can provide guidance on immune checkpoint inhibition (the target is PD-1, PD-L1, CTLA4, etc.), chemotherapy (the typical medicine is gemcitabine), and targeted monoclonal antibody therapy (cusatuzumab, etc.) for treating cancer and autoimmune disease.

Current cancer treatments mainly include surgery, radiotherapy, targeted therapy, and immunotherapy. Among these therapies, immunotherapy has become an important modality in modern cancer treatment by manipulating the autoimmune system to recognize and attack cancer cells [119]. In Figure 4, we concluded the potential therapies strategies for oncology and autoimmune disease. However, the current response rate and efficacy of immunotherapy remain unsatisfactory, which is mainly related to the complex immune escape mechanisms in tumors [280]. Much evidence suggests that m6A methylation can modulate tumor immunity to enhance the efficacy of immunotherapy [17]. MYC is one of the most commonly activated oncogenes mediating cancer initiation and progression, and it regulates the expression of two immune checkpoints, CD47 and PD-L1, by directly binding to their promoters, leading to the identification of MYC as a target for immunotherapy resistance [281]. m6A regulators, including METTL3, FTO, and IGF2BP2, act as tumor promoters through the C-myc pathway in an m6A-dependent manner to promote the growth, invasion, migration, and progression of various tumors, such as BC, lung cancer, and GC [109,282,283]. Wnt/β-catenin signaling promotes chemo- and immunotherapy resistance in various cancers by affecting tumor cell function and immune surveillance, a signaling pathway that can be regulated by m6A modification, which then promotes chemo- and immunotherapy resistance [284]. In breast cancer, YTHDF1 formally influences immunotherapy efficacy through the WNT pathway [109]. In nasopharyngeal carcinoma, TRIM11 is upregulated by a METTL3-mediated m6A modification that regulates β-catenin signaling to promote cisplatin resistance [285]. In addition to the above two pathways, m6A also affects the pathogenesis and progression of cancer through other classical pathways, such as the JAK-STAT1 pathway, ERK/JNK pathway, and NF-κB pathway, which may also provide directions for clinical therapy [55,61,131].

In this comprehensive review, we have outlined the immune regulatory functions and underlying mechanisms of m6A modification across physiological systems and associated cancers and diseases spanning the body, encompassing the respiratory, digestive, endocrine, nervous, urinary systems, and musculoskeletal systems, as well as reproductive system disorders, acute myeloid leukemia, autoimmune conditions, and immune-related diseases. Through the activation of diverse immune cells and catalytic enzymes, m6A modification intricately influences the tumor microenvironment, thereby significantly impacting the onset and progression of various diseases. Moreover, the expression patterns of m6A-related factors also influence disease susceptibility to immunotherapeutic interventions, aiding in the formulation of diagnostic and prognostic models and facilitating the development of novel therapeutic agents.

By synthesizing the pathogenic processes and associated regulatory elements and pathways implicated in m6A modification of immune cells across diverse systemic diseases, we have identified several common threads. Firstly, alterations in the expression levels of m6A writers, readers, and erasers can reshape the tumor microenvironment by directly or indirectly influencing immune cells through mRNA modifications. Previous research has established that the primary role of m6A lies in post-transcriptional fine-tuning and degradation of m6A-tagged transcripts [286]. Secondly, m6A modification can influence the responsiveness to immune checkpoint blockade therapy and the overall prognosis of the disease by modulating the immune microenvironment and regulating the expression of immune checkpoints in immune cells. This underscores the potential for developing m6A-targeted therapeutics and specific m6A inhibitors to complement conventional radiotherapy, chemotherapy, or anti-PD-1 immunotherapy strategies.

While high-throughput techniques have significantly enhanced the detection of m6A modifications within tissue structures across various species, there remains a potential for bias in predicting modification sites and characteristics [287]. Moreover, the comprehensive role of m6A and its associated functions have not been fully elucidated across all immune cell types. Besides m6A, the role of other non-m6A modifications in disease pathogenesis has also garnered increasing attention, such as 2′-O-methylation (Nm), 5-methylcytidine (m5C), N1-methyladenosine (m1A), N7-methylguanosine (m7G), and pseudouridine (ψ) [288,289]. These modifications have a significant impact on evading antiviral immunity, promoting replication, and altering the genetic code of mRNA-coding genes.

Overall, the m6A epitranscriptome marks a new phase in exploring the physiological and pathological mechanisms of immune regulation across diverse systems. Its considerable potential lies in aiding the identification of promising therapeutic targets for diseases, advancing drug discovery efforts and ultimately improving prognostic outcomes.

## Figures and Tables

**Table 1:** Roles of m6A protein machineries and immunological mechanisms in cancers.

System	Cancer Type	m6A Regulator	Immune Cell Type	Target Genes	Mechanism	Immunity Expression	Methods of Treatment	Refs.
Digestive system	Esophageal squamous cell carcinoma (ESCC)	HNRNPC RBM15 IGF2BP3 METTL16 KIAA1429	macrophages, neutrophils			negative		[50]
		YTHDF2 METL14 KIAA1429				negative	Immune-checkpointinhibition, PD-L1	[51]
		YTHDC1 YTHDC2 YTHDC3 ZC3H13	macrophages		HNRNPA2B1——p53 mutation	positive		[52]
		HNRNPA2B1	T cell			positive		[52]
		HNRNPC	B cell			positive	Immune-checkpointinhibition, PD-L1	[52]
	Gastric cancer (GC)	KIAA1429 (recruiting METTL3, METTL14, and WTAP)	DC		C-type lectin receptors, NOD-like receptors, T-cell receptors, toll-like receptors,NF-κB signaling pathways	negative	Immune-checkpointinhibition,PD1/L1	[53,54]
		YTHDF1	recruitment of DCs, CD4+ and CD8+ T cells, adaptive T-cell		FNGR1 and JAK1/2-STAT1 pathway	negative		[55,56]
		ALKBH5	naïve B cells, neutrophils, plasma cells, and follicular helper T cells	SLC7A2, CGB3		positive		[57]
		YTHDF3			PI3K/AKT pathway	positive	Immune-checkpointinhibition, PD-L1,CXCL1	[58,59]
	Liver cancer	ALKBH5	myeloid-derived suppressor-like cells			negative		[60]
			PD-L1+macrophage	MAP3K8	JNK and ERK pathways	positive	Immune-checkpointinhibition,PD-L1	[61]
		YTHDF2	T (Treg) cells		NF-κB signaling	negative		[62,63]
		METTL5	B cells, CD8+ T cells, CD4+ T cells, macrophages, neutrophils and DCs		ribosomal and oxidative phosphorylation, mismatch repair, spliceosome and other signaling pathways	positive	PD1 (PDCD1),CTLA4	[64,65,66]
	Pancreatic cancer	METTL3		lncRNA MALAT1		positive	Immune-checkpointinhibition,PD-L1	[67,68]
		METTL16	B and CD8+ T cell			positive	Immune-checkpointinhibition	[69]
	Colorectal cancer	YTHDF1	CD8+Tcell, T cells and NK cells	IFN-γ-related gene		negative	Immune-checkpointinhibition,PD-1	[70]
			granulocytic myeloid-derived suppressor cells (G-MDSCs), neutrophils		YTHDF1-p65-CXCL1/CXCR2 axis	positive		[71]
		METTL14	dysfunctional T cell			negative		
		METTL3 METTL14	CD8+ T cells	Stat1	IFN-γ-Stat1-Irf1 signaling pathway	negative		[72]
		ALKBH5	CD8+ T cells		NF-κB-CCL5	positive		[73]
		IGF2BP3	NK CD56dim cells, T helper cells, eosinophils, DC, and macrophage neutrophils			positive	Immune-checkpointinhibition,PD-1, CTLA-4	[74,75]
	Gallbladder cancer	YTHDF2		DAPK3		negative	Chemotherapy, Gemcitabine	[76]
Respiratory system	lung adenocarcinoma	LRPPRC	DC, CD8+ cytotoxic T cells, macrophage			negative	Immune-checkpointinhibition,PD-1	[77]
		METTL3	CD8 T cells		activation of chemokine signaling pathways, T cell receptor signaling pathways, and B cell receptor signaling pathway	negative	Immune-checkpointinhibition,PD-1 (Nivoumab)	[78]
		IGF2BP2 CBLL1 HNRNPA2B1	CD4 memory activated T cells, resting NK cells, M0 macrophages, M1 macrophages, and resting mast cells			positive	Immune-checkpointinhibition,LAG3	[79,80]
		YTHDF2 YTHDF3 FTO				negative	Immune-checkpointinhibition, LAG3	[80]
	Nasopharyngeal Carcinoma	LRPPRC	Th2 cell			negative	Immune-checkpointinhibition,PD-L1	[81]
		ALKBH5	NK cells, T cells (CD8+ T and CD4+ T), DCs, M1 macrophages	RIG-I	ALKBH5/RIG-I/IFNα	negative		[82]
Endocrine System	Thyroid cancer	METTL3	CD70 expression and M2 macrophages and Tregs infiltration			negative	Targeted therapy,cusatuzumab	[83]
	Pituitary adenomas	YTHDF2	M2 macrophages			positive	Immune-checkpointinhibition,PD-L1	[84]
Nervous System	Glioblastoma	FTO ZC3H13 YTHDC1	TCM cell			positive		[85]
		FTO ZC3H13	macrophages			negative		[85]
		ALKBH5	CD3, CD4, CD8 T cells			negative	Immune-checkpointinhibition,PD-L1	[86]
		METTL4 WTAP VIRMA	CD8 + T cells, macrophage cells, and myeloid dendritic cells	TFRC		positive	Immune-checkpointinhibition,PD-L1(cd274),CTLA4	[87]
		WTAP	macrophages	HSPA7	YAP1-LOX	positive	Immune-checkpointinhibition,PD-1	[88]
Urinary System	Prostate cancer	METTL14 ZC3H13	Th1 cells, Th17 cells, stromal fraction and TGF-beta response			positive	Immune-checkpointinhibition,PD-1	[39,89]
		HNRNPC	Treg cells			positive	Immune-checkpointinhibition,CTLA-4	[90]
			effector CD8 T cell			negative		[90]
	Kidney renal clear cell carcinoma	METTL14	CD4+ T cells, CD8+ T cells, neutrophils, natural killer (NK) cells, myeloid dendritic cells, macrophages, and B cells		NF-κB pathway	positive	Immune-checkpointinhibition, CD274(PD-L1),HAVCR2,PDCD1LG2(PD-L2),SIGLEC15,TIGIT	[91,92,93]
		METTL14 RBM15B VIRMA ZC3H13 FTO YTHDF2 YTHDC1 HNRNPC1	CD56 bright NK cells, mast cells, NK cells, pDC	CDH13		positive	Immune-checkpointinhibition,PDCD1,CD274,PDCD1LG2,CTLA4,LAG3	[94,95,96]
		METTL5	NK T cell, naïve CD8+T cell, effector memory CD8+ T cell, naïve CD4+ T cell, central memory CD4+ T cell, T helper 1 (Th1) CD4+ T cell, plasma cytoid dendritic cell, myeloid dendritic cell, mast cell, macrophage M2, macrophage M1, macrophage, eosinophil, class-switched memory B cell, plasma B cell, naïve B cell, memory B cell and B cell			negative		[97]
		METTL14 ALKBH5 YTHDC1 RBM15B LRPPRC IGF2BP2 IGF2BP3	NK cells, neutrophils, endothelial cell	ALDOB		positive or negative correlation is unknown		[98]
	Bladder cancer	FTO IGF2BP3	M2 macrophage	PGM1, ENO1		positive		[99]
		YTHDC1	M2 macrophage	PGM1, ENO1		negative		[99]
Reproductive System	Cervical cancer	METTL16 YTHDF1 ZC3H13				negative	Immune-checkpointinhibition,PD-L1	[100]
		IGF2BP3	Treg cells	GLS, GLUD1		positive		[101,102]
		METTL3	CD33+MDSCs			positive		[103]
	Endometrial cancer	METTL14 ZC3H13 YTHDC1	CD4+ Tell			positive	Immune-checkpointinhibition,PD-L1	[104]
			macrophage			negative		[105,106]
	Ovarian cancer		NK, T central memory cells (Tcm) and T gamma delta cells	CDC42EP3		positive		[107,108]
	Breast Cancer	YTHDF1	T cells CD4 memory activated, macrophages M1, NK cells		WNT pathway	positive	Immune-checkpointinhibition	[109]
		HNRNPC VIRMA	CD8 T cells		TFAP2A/DDR1	negative		[110]
Musculoskeletal System	Melanoma	FTO					Immune-checkpointinhibition,PD-1, CXCR4, SOX10	[111,112]
		ALKBH5	Tregs, PMN-MDSCs			negative		[113,114]
			DCs			positive	Immune-checkpointinhibition,PD-1	[113,114]
Acute myeloid leukaemia	METTL14	most immune cells				negative	Immune-checkpointinhibition,CTAL4 (negative)	[115]
		ZC3H13	most immune cells			positive	Immune-checkpointinhibition,CTAL4 (negative)	[115]
		YTHDC2				positive	Immune-checkpointinhibition,PD-L1	[115]
		RBM15				negative	Immune-checkpointinhibition,PD-L1	[115]

**Table 2:** Roles of m6A regulators and immune effects in autoimmune and immune-related diseases.

Autoimmune Disease	m6A Regulator	Immune Cell Type	Immunity Expression	Target Gene	Mechanism	Treatment	Refs.
Rheumatoid arthritis (RA)	METTL3	Macrophage polarization (M1)	positive	STAT1	STAT1 methylation	Sarsasapogenin (Sar)	[41]
	IGF2BP3	Macrophage polarization (M1)	positive	Fibroblast-like synovial cells (FLS)	G2/M transition	Infliximab	[213]
	METTL3	Macrophage	negative		NF-κB signaling pathway		[214]
Systemic lupus erythematosus (SLE)	METTL3	Treg	negative	SOCS	il-2-stat5 signaling pathway		[215,216]
	METTL3	B cell	positive	FOXO3			[217]
	METTL3	CD4+ T cell, Th1 cell, Th17 cell	positive				[218]
	ALKBH5	T cell	positive				[219,220]
Inflammatory bowel disease (IBD)	YTHDC1	Macrophage	positive		Fine-tuned NME nucleoside diphosphokinase 1 (NME1)	Vitolizumab	[221]
	METTL14	T cell	positive		Naïve T cells induce inflammation	Coptisine (COP)	[222]
	FTO	Th17	positive				[223]
Primary Sjögren’s syndrome (pSS)	METTL3	Dentric cell and T cell	positive				[224]
		Mastocyte	positive	FMR1			[225]
ankylosing spondylitis (AS)	METTL14	Mesenchymal stem cells (MSC) migration			The ALKBH5-PRMT6 axis controls the activation of AKT signaling pathway		[226]
	YTHDF2, ALKBH5	Peripheral blood mononuclear cell (PBMC)	negative				[227]
Autoimmune Hepatitis (AIH)	YTHDF2	Bone marrow-derived suppressor cells (MDSCs)	positive				[228]
Autoimmune encephalomyelitis (EAE)	ALKBH5	CD4+ T cell	negative	IFNG, CXCL2	IL-17 signaling pathway		[229]
Allergic respiratory Disease (ARD)	METTL3	M2 macrophage	negative		PI3K/AKT and JAK/STAT6 signaling pathway	Methylprednisolone, Chelidonine, Nerabine, Ribavirin	[230]
HIV	YTHDF3	CD4+ T cell	negative		Increasing HIV-1 reverse transcription	Protease inhibitors (indinavir, amprenavir, etc.)	[231,232,233]
